# Critical Resources for Hospital Surge Capacity: An Expert Consensus Panel

**DOI:** 10.1371/currents.dis.67c1afe8d78ac2ab0ea52319eb119688

**Published:** 2013-10-07

**Authors:** Jamil D. Bayram, Lauren M. Sauer, Christina Catlett, Scott Levin, Gai Cole, Thomas D. Kirsch, Matthew Toerper, Gabor Kelen

**Affiliations:** Department of Emergency Medicine, School of Medicine, Johns Hopkins University, Baltimore, Maryland, USA; Johns Hopkins Office of Critical Event Preparedness and Response, Baltimore, Maryland, USA; Center for Refugee and Disaster Response, Bloomberg School of Public Health, Johns Hopkins University, Baltimore, Maryland, USA; Department of Emergency Medicine, School of Medicine, Johns Hopkins University, Baltimore, Maryland, USA; Johns Hopkins Office of Critical Event Preparedness and Response, Baltimore, Maryland, USA; Center for Refugee and Disaster Response, Bloomberg School of Public Health, Johns Hopkins University, Baltimore, Maryland, USA; Department of Emergency Medicine, School of Medicine, Johns Hopkins University, Baltimore, Maryland, USA; Johns Hopkins Office of Critical Event Preparedness and Response, Baltimore, Maryland, USA; Department of Emergency Medicine, School of Medicine, Johns Hopkins University, Baltimore, Maryland, USA; Department of Emergency Medicine, School of Medicine, Johns Hopkins University, Baltimore, Maryland, USA; Johns Hopkins Office of Critical Event Preparedness and Response, Baltimore, Maryland, USA; Department of Emergency Medicine, School of Medicine, Johns Hopkins University, Baltimore, Maryland, USA; Johns Hopkins Office of Critical Event Preparedness and Response, Baltimore, Maryland, USA; Center for Refugee and Disaster Response, Bloomberg School of Public Health, Johns Hopkins University, Baltimore, Maryland, USA; Department of Emergency Medicine, School of Medicine, Johns Hopkins University, Baltimore, Maryland, USA; Department of Emergency Medicine, School of Medicine, Johns Hopkins University, Baltimore, Maryland, USA; Johns Hopkins Office of Critical Event Preparedness and Response, Baltimore, Maryland, USA

## Abstract

Background: Hospital surge capacity (HSC) is dependent on the ability to increase or conserve resources. The hospital surge model put forth by the Agency for Healthcare Research and Quality (AHRQ) estimates the resources needed by hospitals to treat casualties resulting from 13 national planning scenarios. However, emergency planners need to know which hospital resource are most critical in order to develop a more accurate plan for HSC in the event of a disaster. Objective: To identify critical hospital resources required in four specific catastrophic scenarios; namely, pandemic influenza, radiation, explosive, and nerve gas. Methods: We convened an expert consensus panel comprised of 23 participants representing health providers (i.e., nurses and physicians), administrators, emergency planners, and specialists. Four disaster scenarios were examined by the panel. Participants were divided into 4 groups of five or six members, each of which were assigned two of four scenarios. They were asked to consider 132 hospital patient care resources- extracted from the AHRQ's hospital surge model- in order to identify the ones that would be critical in their opinion to patient care. The definition for a critical hospital resource was the following: absence of the resource is likely to have a major impact on patient outcomes, i.e., high likelihood of untoward event, possibly death. For items with any disagreement in ranking, we conducted a facilitated discussion (modified Delphi technique) until consensus was reached, which was defined as more than 50% agreement. Intraclass Correlation Coefficients (ICC) were calculated for each scenario, and across all scenarios as a measure of participant agreement on critical resources. For the critical resources common to all scenarios, Kruskal-Wallis test was performed to measure the distribution of scores across all scenarios. Results: Of the 132 hospital resources, 25 were considered critical for all four scenarios by more than 50% of the participants. The number of hospital resources considered to be critical by consensus varied from one scenario to another; 58 for the pandemic influenza scenario, 51 for radiation exposure, 41 for explosives, and 35 for nerve gas scenario. Intravenous crystalloid solution was the only resource ranked by all participants as critical across all scenarios. The agreement in ranking was strong in nerve agent and pandemic influenza (ICC= 0.7 in both), and moderate in explosives (ICC= 0.6) and radiation (ICC= 0.5). Conclusion: In four disaster scenarios, namely, radiation, pandemic influenza, explosives, and nerve gas scenarios; supply of as few as 25 common resources may be considered critical to hospital surge capacity. The absence of any these resources may compromise patient care. More studies are needed to identify critical hospital resources in other disaster scenarios.

## Introduction

Hospital surge capacity is dependent on the ability to increase or conserve resources, in response to an influx of patients in a disaster situation^ 1^. Adequate surge capacity depends on the fundamental understanding of which hospital resources are critical to patient care 2. Surge capacity has four conceptual components, three of which categorize resources; space, staff, and supplies^ 3^^,^ 4 ^,^ 5. The fourth key component defining surge capacity is the “system” category, which denotes organization, processes, policies and procedures that govern and organize the allocation and conservation of the first three components.

In 2009, the Agency for Healthcare Research and Quality (AHRQ) developed a Hospital Surge Model 6 that forecasted the hospital resources required to treat casualties resulting from 13 National Planning Scenarios^ 7^. The AHRQ's planning tool was discontinued on June 30, 2011^6^. The objective of our study was to identify which of the AHRQ hospital resources were the most critical to care for patients in four of the National Planning Scenarios: pandemic influenza, radiation event, explosion, and nerve gas attack 7. This information is essential for hospital preparedness planners to make supply-chain driven decisions based on the number of patients treated. In addition, we wished to determine which of these critical hospital resources were common across multiple types of events, and distinguish resources that are critical only for specific scenarios.

## Methods

We organized and conducted a facilitated national expert consensus panel held during the spring of 2011. Twenty three expert panelists from various disciplines in the health care sector (Table 1) took part in a one day consensus-building set of exercises. The majority of the participants were purposefully chosen to be healthcare providers, because the decision of what constitutes critical hospital resource for patient care, and issues related to standards of care are primarily clinical. However, we did include allied professionals to ensure other perspectives. The study was approved by an Institutional Review Board on Human Subjects Research, and all study participants provided appropriate consent.

**Table 1. Expert panel composition:** (in alphabetical order)

**Participant Type**	**Number** ****
Administration and hospital operations	4
Adult intensive care nurse	2
Adult intensive care physician	2
Burn specialist	1
Infectious disease physician	1
Internal medicine physician	2
Medical/surgical nurse	2
Pediatric intensive care nurse	1
Pediatric intensive care physician	2
Pediatric nurse	2
Pediatric physician	2
Radiation specialist	1
Trauma physician	1

**Prior to the exercises, the investigators conducted a detailed review of the literature and produced a list of all patient care related resources (space, staff, supplies) that may be required for routine use and for specific disaster scenarios based on four of the national planning scenarios chosen for these exercises *(pandemic influenza scenario, radiation exposure, explosives, and nerve gas)*. The radiation scenario postulates a radiological dispersal device or “dirty bomb” detonated in 3 different sites moderate-to-large cities causing 180 fatalities; 270 injuries; 20,000 detectable contaminations (at each site). The pandemic influenza scenario posits a 15% attack rate: 87,000 fatalities; and 300,000 hospitalizations. In the nerve gas scenario, six spray dissemination devices and releases Sarin vapor into the ventilation systems of three large commercial office buildings in a metropolitan area causing 6,000 fatalities (95% of building occupants). In the explosion scenario, improvised explosive devices (IEDs) were detonated a sports arena, an underground public transportation concourse, and in a parking facility near the entertainment complex causing 100 fatalities and 450 hospitalizations.

Participants were provided key background literature, as well as the URL to the original AHRQ model, and a list of the resources to review two weeks prior to the meeting. The finalized resource list was provided to all participants at a meeting held the evening prior to the day of the exercises. To ensure uniform application of the planned methods the next day, two separate mock exercises (using different national planning scenarios) were conducted at the evening session with the entire group of expert panelists. During that session, participants were also briefed on the logistics of the consensus panel the following day. On the day of the formal exercises, participants were divided into 4 groups of five or six members. Each group was assigned two of the four scenarios. , i.e. each of the four scenarios was assigned to two separate groups, in various combinations. Each group had a facilitator to guide the proceedings and discussion. Weeks prior to the study, the PI and the Project Manager chose and trained four experienced facilitators. Panelists in each group were asked to consider 132 hospital patient care resources – extracted from the AHRQ's hospital surge model and the literature – in order to identify the ones that would be critical to patient care. Both pediatric and adult patients were to be considered. The operational definition of a "critical" hospital resource was as follows: *absence of the resource is likely to have a major impact on patient outcomes, i.e., high likelihood of untoward event, possibly death.*

By design, morbidity, mortality and the number of victims for various scenarios have no bearing on the outcomes of interest since the main objective of the study was to determine which specific hospital resources were considered critical to care for patients of all levels of acuity, regardless of the clinical load. In this regard, two major assumptions were communicated to the panelists. First, they were asked to assume that basic hospital/unit operations, such as infrastructure, food, security, housekeeping, laundry, etc., remained intact, and that the hospital building was not degraded structurally in any way. Second, it was assumed that clinical standards of care would be maintained, i.e. panelists were asked to perform the exercise without considering the availability of alternate or compensatory resources that did not maintain equivalent standards of care. The PI of the study and the Project Manager continuously rotated among panels to clarify definitions and methods, and to ensure that each panel was adhering to the protocols, but did not espouse any particular point of view.

For items with any disagreement in ranking within each group, a facilitated discussion (modified Delphi technique) was conducted until consensus (defined as more than 50% agreement) was reached. To help mitigate potential dominance by one or more panel members, rankings by each individual panelist was done separately and in a blinded fashion. Each group was also encouraged to add potential hospital resources not already on the list. Panel members were asked to recuse themselves from exercising an opinion and score on resource items with which they were not familiar.

**Analysis**

To measure the agreement among all participants on assigning critical resources, intraclass correlation coefficient (ICC) for every item was calculated individually for each scenario and collectively across all scenarios; Kruskal-Wallis test was performed to measure the distribution of scores for each resource across all scenarios.

**Ethics Statement**

This work was reviewed and approved by the Johns Hopkins School of Medicine Institutional Board and a waiver of consent was approved and documented by the Program Manager. The waiver was given because the research involves no risk to participants as they were invited to participate and could choose at any time not to attend. They were offering their opinion on a subject they are experts in and all of their opinions and all related data were recorded unidentifiable and anonymously. By recording their consent, we would be introducing identifiers.

## Results

Of the 132 hospital resources evaluated, 25 were considered critical in all four scenarios by more than 50% of participants (Table 2). There was 90% or more agreement among panelists on 16 of these 25 hospital resources, with agreement ranging from 64.7% to 100%. Crystalloid solution was the only resource that had 100% agreement on being critical, in all four scenarios.

**Table 2 -Critical hospital resources common to all four scenarios:** *Significant difference in score distribution among the four scenarios

**** **Resource**	**** **Percent scored as 3 (Critical)**	**** **p-value (Kruskal-Wallis)** **** ****
Crystalloid solution with IV tubing	100	1
Adult ICU capacity	97.8	0.364
Ambu bag, adult	97.8	0.364
Endotracheal tube	97.8	0.364
Laryngoscope, adult	97.8	0.364
Oxygen source and tubing	97.8	0.364
Ambu bag, pediatric	95.7	0.526
Adult mechanical ventilator set	95.7	0.089
Pediatric mechanical ventilator set	95.7	0.089
Critical care nurse	95.7	0.526
Suction catheter and suction apparatus	95.7	0.089
Laryngoscope, peds	95.7	0.561
Critical care physician	93.5	0.803
Sedatives*	93.5	0.031
Peds ICU capacity	93.5	0.775
Adult medical/surgical bed	91.3	0.56
Needles, sterile	80.4	0.166
Non-critical care nurse	80.4	0.234
Latex-free, non-sterile gloves*	78.3	0.036
IV catheters (18-24g), and heplocks	78.3	0.158
Pressors*	76.1	0.028
BP cuffs, adult	71.7	0.078
BP cuffs, pediatric	71.7	0.072
Peds medical/surgical bed	67.4	0.21
Oxygen mask, adult	67.4	0.068

Out of the 132 hospital resources, the number considered critical by more than 50% of participants varied from one scenario to another; 58 (44%) for the pandemic influenza scenario, 51 (39%) for radiation exposure, 41 (31%) for explosives, and 35 (27%) for nerve gas scenario (Table 3). There were an additional 10 scenario-specific hospital resources considered critical by all participants (100% agreement), that were not considered as generally critical for all scenarios. These 10 resources were distributed as follows: 5 in the pandemic influenza scenario (isolation room/cohorting, respiratory therapist, facemasks, antiviral agents for influenza, and dialysis); 4 in the radiation scenario (radiation specialist, potassium iodide, Geiger counter, and decontamination capability); 1 in the explosives scenario (standard radiograph), and none specific for nerve agents. There were a number of resources that were specific to a single scenario (Table 3) as follows: pandemic influenza (isolation room/cohorting, respiratory therapist, facemasks, antiviral agents for influenza, and dialysis); radiation (radiation specialist, potassium iodide, Geiger counter, decontamination capability and isotope chelating agents); explosion (surgeon, packed red blood cells, Silvadene cream, gauze pads, fresh-frozen plasma, enteral feeding tubes); and nerve agent (Atropine and 2-PAM).

Agreement on all 132 resources was strong for the pandemic influenza (ICC= 0.7, CI: 0.624 to 0.746) and nerve gas scenarios (ICC=0.7, CI:0.636 to 0.753), and moderate for explosives (ICC=0.6, CI: 0.555 to 0.686) and radiation exposure scenarios (ICC=0.5, CI: 0.435 to 0.588). In general, there was similarity in the distribution (i.e. no significant difference; p>0.05) of the identified 25 critical hospital resources across all four scenarios (Table 2), with the exception of resources: sedatives (p=0.031), vasopressors (p=0.036), and non-sterile latex-free gloves (p=0.028).

**Table 3 - Scenario-Specific Critical Hospital Resources:** 

**Pandemic Influenza**		**Radiation**		**Explosives**		**Nerve Gas**	
**Resource**	**% ranked as critical**	**Resource**	**% ranked as critical**	**Resource**	**% ranked as critical**	**Resource**	**% ranked as critical**
1. Crystalloid solution (NS or LR) IV, 1000 ml, and IV tubing*	100	1. Crystalloid solution (NS or LR) IV, 1000 ml, and IV tubing	100	1. Crystalloid solution (NS or LR) IV, 1000 ml, and IV tubing	100	1. Crystalloid solution (NS or LR) IV, 1000 ml, and IV tubing	100
2. Adult ICU capacity	100	2. Decontamination capability	100	2. Adult ICU capacity	100	2. Peds ICU capacity	100
3. ICU MD	100	3. Potassium iodide	100	3. Critical care nurse (CCN)	100	3. Critical care nurse (CCN)	100
4. Respiratory Therapist (RT)	100	4. Radiation expert	100	4. Pressors	100	4. Sedatives	100
5. Case Specific Antibiologic (ex - Antivirals for PanFlu)	100	5. Geiger counter	100	5. Adult med/surg bed	100	5. Adult ICU capacity	100
6. Sedatives	100	6. Sedatives	100	6. Suction catheter and suction apparatus	100	6. Suction catheter and suction apparatus	100
7. IV catheters, small bore (18-24g), and heplock	100	7. Standard radiograph	90.9	7. Adult mechanical ventilator set	100	7. Adult mechanical ventilator set	100
8. Airborne isolation room or cohorting	100	8. Isotope chelating agents	90.9	8. Pediatric mechanical ventilator set	100	8. Pediatric mechanical ventilator set	100
9. Suction catheter and suction apparatus	100	9. Ambu bag, adult	90.9	9. Endotracheal tube	100	9. Endotracheal tube	100
10. Adult mechanical ventilator set	100	10. Ambu bag, pediatric	90.9	10. Laryngoscope, adult	100	10. Laryngoscope, adult	100
11. Pediatric mechanical ventilator set	100	11. Oxygen mask, adult (any)	90.9	11. Ambu bag, adult	100	11. Laryngoscope, peds	100
12. Endotracheal tube	100	12. NG tubes	90.9	12. Oxygen (O2) source and tubing	100	12. Ambu bag, adult	100
13. Laryngoscope, adult	100	13. BP cuffs, adult	90.9	13. Standard radiograph	100	13. Ambu bag, pediatric	100
14. Laryngoscope, peds	100	14. BP cuffs, pediatric	90.9	14. ICU MD	91.7	14. Oxygen (O2) source and tubing	100
15. Ambu bag, adult	100	15. Adult med/surg bed	90.9	15. Laryngoscope, peds	91.7	15. ICU MD	91.7
16. Ambu bag, pediatric	100	16. Adult ICU capacity	90.9	16. Ambu bag, pediatric	91.7	16. Pulse oximeter	91.7
17. Oxygen (O2) source and tubing	100	17. Peds ICU capacity	90.9	17. Needles, sterile	91.7	17. Cardiac monitor	91.7
18. Face shields	100	18. ICU MD	90.9	18. Peds ICU capacity	91.7	18. Decontamination capability	91.7
19. Latex-free, non-sterile gloves	100	19. Broad spectrum abx	90.9	19. Surgeon	91.7	19. Chem/rad PPE for staff	91.7
20. Dialysis capability	100	20. FFP	90.9	20. Narcotic pain medication	91.7	20. Adult med/surg bed	83.3
21. Peds med/surg bed	90.9	21. IV catheters, large bore (14-16g), and heplock	90.9	21. Packed RBCs	91.7	21. Latex-free, non-sterile gloves	83.3
22. Peds ICU capacity	90.9	22. Bed linen	90.9	22. Non-critical care nurse (RN/LPN)	83.3	22. Atropine	83.3
23. Bronchodilators	90.9	23. Dialysis capability	90.9	23. Pulse oximeter	83.3	23. 2-PAM	83.3
24. Needles, sterile	90.9	24. Critical care nurse (CCN)	90.9	24. Sedatives	75	24. Syringes (3cc-10cc)	75
25. Temperature monitor, adult	90.9	25. IV Pump	90.9	25. Broad spectrum abx	75	25. IV catheters, small bore (18-24g), and heplock	66.7
26. Temperature monitor, pediatric	90.9	26. Endotracheal tube	90.9	26. IV catheters, small bore (18-24g), and heplock	66.7	26. Peds med/surg bed	58.3
27. Standard radiograph	90.9	27. Laryngoscope, adult	90.9	27. Trauma bandages	66.7	27. Non-critical care nurse (RN/LPN)	58.3
28. Broad spectrum abx	90.9	28. Laryngoscope, peds	90.9	28. IV catheters, large bore (14-16g), and heplock	66.7	28. Needles, sterile	58.3
29. N-95 masks	90.9	29. Oxygen (O2) source and tubing	90.9	29. Syringes (3cc-10cc)	58.3	29. BP cuffs, adult	58.3
30. Adult med/surg bed	90.9	30. Internist/Hospitalist	90.9	30. Cardiac monitor	50	30. BP cuffs, pediatric	58.3
31. Critical care nurse (CCN)	90.9	31. Non-critical care nurse (RN/LPN)	90.9	31. Peds med/surg bed	50	31. Pressors	50
32. IV catheters, large bore (14-16g), and heplock	90.9	32. Adult mechanical ventilator set	81.8	32. BP cuffs, adult	50	32. Oxygen mask, adult (any)	50
33. Pressors	90.9	33. Pediatric mechanical ventilator set	81.8	33. Latex-free, non-sterile gloves	50	33. Defibrilator	50
34. Chest tube drainage system	90.9	34. Platelets	81.8	34. Oxygen mask, adult (any)	50	34. Morgue space	50
35. BP cuffs, adult	90.9	35. Packed RBCs	81.8	35. Chest tube drainage system	50	35. Bed linen	50
36. BP cuffs, pediatric	90.9	36. IV catheters, small bore (18-24g), and heplock	81.8	36. BP cuffs, pediatric	50		
37. Gown, standard	90.9	37. Latex-free, non-sterile gloves	81.8	37. Silvadene cream	50		
38. Non-critical care nurse (RN/LPN)	90.9	38. Pharmacist (PharmD/RPh)	81.8	38. Gauze pad (2x2 or 4x4)	50		
39. Pharmacist (PharmD/RPh)	81.8	39. Narcotic pain medication	81.8	39. FFP	50		
40. IV Pump	81.8	40. Needles, sterile	81.8	40. Enteral feeding tube	50		
41. Nasal cannula	81.8	41. Suction catheter and suction apparatus	81.8	41. Morgue space	50		
42. Oxygen mask, adult (any)	81.8	42. Peds med/surg bed	72.7				
43. Surgical masks	81.8	43. Alcohol hand sanitizer	72.7				
44. Alcohol hand sanitizer	81.8	44. Antiemetics	72.7				
45. Bed linen	81.8	45. Temperature monitor, pediatric	63.6				
46. Pulse oximeter	72.7	46. Pressors	63.6				
47. Internist/Hospitalist	72.7	47. D5W or sterile water piggybacks, 50-250ml	54.5				
48. Cardiac monitor	63.6	48. Defibrilator	54.5				
49. ID specialist	54.5	49. Chem/rad PPE for staff	54.5				
50. Paralytics	54.5	50. Temperature monitor, adult	54.5				
51. Defibrilator	54.5	51. Personal dosimeters	54.5				
52. NG tubes	54.5						
53. Steroids	54.5						
54. Narcotic pain medication	54.5						
55. Syringes (3cc-10cc)	54.5						
56. D5W or sterile water piggybacks, 50-250ml	54.5						
57. Bleach-based cleaner	54.5						
58. Gram (+) Abx	54.5						

## Discussion

In the face of natural and man-made disasters, hospitals are at the forefront providing medical care for patients at all levels of acuity. While there has been considerable work related to surge capacity 1 ^,^ 2 ^,^ 3 ^,^ 4 ^,^ 5 ^,^ 8 ^,^ 9 ^,^ 10 ^,^ 11 ^,^ 12 ^,^ 13 ^,^ 14 ^,^ 15 ^,^ 16 ^,^ 17 ^,^ 18 ^,^ 19 ^,^ 20 ^,^ 21 ^,^ 22 ^,^ 23 ^,^ 24 ^,^ 25 ^,^ 26 ^,^ 27 ^,^ 28 ^,^ 29 ^,^ 30 ^,^ 31 ^,^ 32 ^,^ 33 ^,^ 34 ^,^ 35 , and national recommendations to itemize critical healthcare resources 35 , detailed accounting of individual hospital resources that are critical for response to specific types of disasters is lacking. Hospital administrators and disaster managers must identify the critical medical resources in all likely scenarios, and design robust storage and supply-chain protocols to maintain adequate supplies of these resources.

In 2009, Hick *et al*. categorized surge capacity according to three levels of graded response: “conventional,” “contingent,” or “crisis” level management. [2] A conventional response implies that routinely available resources can address the requirements imposed by the event, and standards of care can be maintained. Contingent response requires mobilization of additional measures to those routinely in place, while standards of care are essentially maintained. Crisis response requires considerable alteration in routine operations and available measures to meet the patient surge requirements, and standards of care cannot be maintained. We believe our study builds upon the model proposed by Hick *et al*., by identifying resources considered critical within the boundaries of conventional and contingency surge capacities.

The 25 resources identified as critical – by consensus – for all 4 scenarios (pandemic influenza, radiation, explosives, and nerve agents) cover the three categories of hospital surge capacity resources; supplies (18), space (4), and staff (3). In the supplies category, intravenous crystalloid solution was the only resource unanimously ranked by participants as critical in all 4 scenarios. The rationale behind this categorization may be related to the ubiquitous use of crystalloid solutions in acute medical conditions resulting from various disaster scenarios. Other common supplies with consensus as being “critical,” include intubation equipment, ventilators, oxygen, sedatives, IV catheters, needles, gloves, and blood pressure cuffs. As expected, some resources are highly specific and critical for response to a certain event. For example, for a disaster with radiation exposure, potassium iodide and a radiation expert were considered critical by 100% of the participants. In the space category, adult and pediatric intensive care and medical/surgical beds were identified as critical resources for all four scenarios. These represent the main physical areas accommodating critical and moderately injured patients requiring admission. In the staff category, critical care physicians and nurses, in addition to non-critical care nurses, were considered critical across all scenarios. Interestingly, non-critical care physicians (e.g. internal medicine physicians) were considered critical by less than 50% of panelists in all four study scenarios. We note that our study panelists placed emphasis on specialized care in disaster response. This is understandable given the orientation of health care practice in the U.S., even during disasters. However, in many countries, primary care resources may play a larger role and would likely be judged as reaching critical hospital resource status in many disaster scenarios.

The differences in agreement among participants regarding all 132 resources, when considering specific scenarios, may be grounded in the degree of clinical and operational familiarity with the specific scenario in question. For example, influenza epidemics occur regularly, so both clinicians and hospital administrators are more familiar with the required critical resources (ICC=0.7),than those needed in a radiation disaster (ICC=0.5). The other interesting finding of variation in the number of critical resources among different scenarios (e.g. 58 for pandemic influenza and 35 for nerve gas), is likely due to the clinical complexity of the scenario, in addition to the notion of familiarity mentioned above.

To the best of our knowledge, the derivation and importance ranking of 132 discrete potential hospital resources, is the only collective attempt by subject matter experts to stratify the various hospital-based provisions required for the care of victims during the four scenarios considered in this study. The expert panelists noted which of these hospital resources were critical, and that their absence represented a major risk of an untoward medical outcome. This information enables planners to further examine, describe and delineate their surge capacity beyond simple bed availability.

**Limitations**

There are several limitations to our study. First, the study is based on an expert panel consensus and not on a specific functional exercise or disaster event. Second, the size of each group (sub-panel) of experts was not very large (five or six members). Still, two separate panels, i.e., 50% of the available panel members, considered each scenario. Third, group composition may not be comprehensive in representing the numerous specialties and sub-specialties that would typically take part in responding to the selected disaster scenarios. Representation from every type of hospital may also be lacking (e.g. rural hospitals were poorly represented). While we were thoughtful and deliberate regarding the composition of each group – seeking to optimize representation from a broad national pool of expert practitioners – this limitation may affect the generalizability of our finding. Finally, due to the objectives of the study, the results are applicable to only four national planning scenarios. To mitigate this limitation, we are planning on conducting further studies to explore identifying critical resources for the remaining national planning scenarios.

**Conclusion**

Twenty five hospital resources were found to be critical to maintain continuity medical care in four disaster planning scenarios; namely radiation, pandemic influenza, explosives, and nerve gas scenarios. However, some specific disaster scenarios require additional critical specialized resources necessary for the corresponding type of disaster. Planning for each hospital should be dictated by the hazard vulnerability analysis, gauging their vulnerabilities within the environment, in order to prioritize and maintain adequate supplies of scenario-specific critical hospital resources. Further studies are needed in the field of hospital surge capacity, to validate these findings, determine utilization rates for each of the resources during a surge event, and to identify appropriate alternatives to these critical hospital resources^36^.

## Competing Interests

The authors have declared that no competing interests exist and have no disclosures.

## References

[1] Kelen GD, McCarthy ML. The science of surge. Acad Emerg Med. 2006; 13:1089–1094. 10.1197/j.aem.2006.07.016 17085736

[2] Hick JL, Barbera JA, Macintyre AG, Kelen GD. Refining surge capacity: conventional, contingency, and crisis capacity. Disaster Med Public Health Preparedness. 2009; 3:S59–S67 10.1097/DMP.0b013e31819f1ae2 19349869

[3] Kaji A, Koenig KL, Bey T. Surge capacity for healthcare systems: a conceptual framework. Acad Emerg Med. 2006; 13:1157–1159. 10.1197/j.aem.2006.06.032 16968688

[4] Barbisch D, Koenig KL. Understanding surge capacity: essential elements. Acad Emerg Med. 2006; 13:1098–1102. 10.1197/j.aem.2006.06.041 17085738

[5] Hick JL, Koenig KL, Barbisch D, et al. Surge capacity concepts for health care facilities: the CO-S-TR model for initial incident assessment. Disaster Med Public Health Preparedness. 2008; 2:S51–S57. 10.1097/DMP.0b013e31817fffe8 18769268

[6] Agency for Healthcare Research on Quality (AHRQ). Hospital Surge Model. Accessed on January 16, 2012.

[7] Federal Emergency Management Agency (FEMA). National Planning Scenarios Factsheet. Accessed on January 16, 2012.

[8] Runge JW. Surge Medical Response Capability: What is it? How do we get it? How do we know when we have it? A white paper prepared for the June 10-11, 2009 workshop on medical surge capacity hosted by the Institute of Medicine Forum on Medical and Public Health Preparedness for Catastrophic Events.

[9] Kelen GD, Kraus CK, McCarthy ML, et al. Inpatient disposition classification for the creation of hospital surge capacity: a multiphase study. Lancet. 2006; 368:1984–1990. 10.1016/S0140-6736(06)69808-5 PMC7138047 17141705

[10] Hick JL, Hanfling D, Burstein JL, et al. Health care facility and community strategies for patient care surge capacity. Ann Emerg Med 2004; 44:253–61. 10.1016/j.annemergmed.2004.04.011 PMC7118880 15332068

[11] Schultz CH, Stratton SJ. Improving hospital surge capacity: a new concept for emergency credentialing of volunteers. Ann Emerg Med. 2007; 49:602–609. 10.1016/j.annemergmed.2006.10.003 17112633

[12] Davis DP, Poste JC, Hicks T, et al. Hospital bed surge capacity in the event of a mass-casualty incident. Prehosp Disaster Med. 2005; 20:169–176. 10.1017/s1049023x00002405 16018505

[13] Soremukun OA, Zane RD, Walls A, Allen MB, Seefeld KJ, Pallin DJ: Cancellation of scheduled procedures as a mechanism to generate hospital bed surge capacity–A pilot study. Prehosp Disaster Med 2011; 26(3):1–6. 10.1017/S1049023X11006248 22107776

[14] Burkle FM Jr. Population-based triage management in response to surge capacity requirements during a large-scale bioevent disaster. Acad Emerg Med. 2006; 13(11):1118–1129. 10.1197/j.aem.2006.06.040 17015415

[15] Estacio PL. Surge capacity for health care systems: early detection, methodologies, and process. Acad Emerg Med. 2006; 13(11):1135–1137. 10.1197/j.aem.2006.07.018 17085739

[16] McManus J, Huebner K, Scheulen J. The science of surge: detection and situational awareness. Acad Emerg Med. 2006; 13(11):1179–1182. 10.1197/j.aem.2006.06.038 16968687

[17] Stratton SJ, Tyler RD. Characteristics of medical surge capacity demand for sudden-impact disasters. Acad Emerg Med. 2006; 13(11):1193–1197. 10.1197/j.aem.2006.05.008 16885401

[18] Bonnett CJ, Peery BN, Cantrill SV, et al. Surge capacity: a proposed conceptual framework. Am J Emerg Med. 2007; 25(3):297–306. 10.1016/j.ajem.2006.08.011 17349904

[19] Hanfling D. Equipment, supplies, and pharmaceuticals: how much might it cost to achieve basic surge capacity? Acad Emerg Med. 2006; 13(11):1232–1237. 10.1197/j.aem.2006.03.567 16801633

[20] Burt CW, McCaig LF. Staffing, capacity, and ambulance diversion in emergency departments: United States, 2003-04. Adv Data. 2006; 27(376):1–23. 17037024

[21] Voelker R. Mobile hospital raises questions about hospital surge capacity. JAMA. 2006; 295(13):1499–1503. 10.1001/jama.295.13.1499 16595745

[22] Koenig KL, Kelen G. Proceedings of the Consensus Conference “The Science of Surge,” May 17, 2006, San Francisco, California, USA. Acad Emerg Med. 2006; 13:1087–1088. 10.1197/j.aem.2006.07.008 17032943

[23] Eastman AL, Rinnert KJ, Nemeth IR, Fowler RL, Minei JP. Alternate site surge capacity in times of public health disaster maintains trauma center and emergency department integrity: Hurricane Katrina. J Trauma. 2007; 63(2):253–257 10.1097/TA.0b013e3180d0a70e 17693820

[24] McCarthy ML, Aronsky D, Kelen GD. The measurement of daily surge and its relevance to disaster preparedness. Acad Emerg Med. 2006; 13(11):1138–1141. 10.1197/j.aem.2006.06.046 17032946

[25] Davis DP, Poste JC, Hicks T, Polk D, Rymer TE, Jacoby I. Hospital bed surge capacity in the event of a mass-casualty incident. Prehosp Disaster Med. 2005; 20(3):169–176 10.1017/s1049023x00002405 16018505

[26] DeLia D. Annual bed statistics give a misleading picture of hospital surge capacity. Ann Emerg Med. 2006; 48(4): 384–388. 10.1016/j.annemergmed.2006.01.024 16997673

[27] Challen K, Walter D. Accelerated discharge of patients in the event of a major incident: observational study of a teaching hospital. BMC Public Health. 2006; 6:108. 10.1186/1471-2458-6-108 PMC1468403 16638157

[28] Roccaforte JD, Cushman JG. Disaster preparedness, triage, and surge capacity for hospital definitive care areas: optimizing outcomes when demands exceed resources. Anesthesiol Clin. 2007; 25(1):161–177. 10.1016/j.anclin.2007.01.002 PMC7185660 17400163

[29] Phillips S. Current status of surge research. Acad Emerg Med. 2006; 13(11):1103–1108. 10.1197/j.aem.2006.07.007 17032944

[30] Asplin BR, Flottemesch TJ, Gordon BD. Developing models for patient flow and daily surge capacity research. Acad Emerg Med. 2006; 13(11):1109–1113. 10.1197/j.aem.2006.07.004 17015412

[31] Rothman RE, Hsu EB, Kahn CA, Kelen GD. Research priorities for surge capacity. Acad Emerg Med. 2006; 13(11):1160–1168 10.1197/j.aem.2006.07.002 17032947

[32] Jenkins JL, O’Connor RE, Cone DC. Differentiating large-scale surge versus daily surge. Acad Emerg Med. 2006; 13(11):1169–1172 10.1197/j.aem.2006.07.022 17085741

[33] Davidson SJ, Koenig KL, Cone DC. Daily patient flow is not surge: “management is prediction.” Acad Emerg Med. 2006; 13(11):1095–1096. 10.1197/j.aem.2006.07.021 17085737

[34] Bayram JD, Zuabi S, Subbarao I. Disaster metrics: quantitative benchmarking of hospital surge capacity in trauma-related multiple casualty events. Disaster Med Public Health Prep. 2011 Jun; 5(2):117–24. Epub 2010 Sep 28. 10.1001/dmp.2010.19 21685307

[35] National guidance for healthcare system preparedness. Assistant Secretary for Preparedness and Response, Department of Health and Human Services. Healthcare preparedness capabilities- Washington, DC. 2012. Accessed May 22, 2013

[36] Olympia RP, Rivera R, Heverley S, Anyanwu U, Gregorits M. Natural disasters and mass-casualty events affecting children and families: a description of emergency preparedness and the role of the primary care physician. Clin Pediatr. 2010 Jul;49(7):686-98. Epub 2010 Mar 31. 10.1177/0009922810364657 20356922

